# Microbiological and clinical characteristics of *Streptococcus gallolyticus subsp. pasteurianus* infection in China

**DOI:** 10.1186/s12879-019-4413-5

**Published:** 2019-09-09

**Authors:** Yi Li, Xingchun Chen, Zhijun Zhang, Lijun Wang, Junrui Wang, Ji Zeng, Junwen Yang, Binghuai Lu

**Affiliations:** 1 Department of Laboratory Medicine, Henan Provincial People’s Hospital, Zhengzhou, 450003 China; 2 Department of Laboratory Medicine, People’s Hospital of Guangxi Zhuang Autonomous Region, Nanning, 530021 China; 3 Department of Laboratory Medicine, Tai’an City Central Hospital (Tai’an), Shandong, 271016 China; 4 Department of Laboratory Medicine, Beijing Tsinghua Chang Gung Hospital, Tsinghua University, Beijing, 102218 China; 5 Department of clinical laboratory, Affiliated hospital of Inner Mongolia medical university, Hohhot, 010050 China; 6 Department of Laboratory Medicine, Wuhan Pu Ai Hospital of Huazhong University of Science and Technology, Wuhan, 430034 China; 7 Department of Laboratory Medicine, Zhengzhou children’s hospital, Zhengzhou, 450018 China; 8 Laboratory of Clinical Microbiology and Infectious Diseases, Department of Pulmonary and Critical Care Medicine, China-Japan Friendship Hospital, No 2, East Yinghua Road, Chaoyang District, Beijing, 100029 China; 9 Center for Respiratory Diseases, China-Japan Friendship Hospital, No 2, East Yinghua Road, Chaoyang District, Beijing, 100029 China; 10 National Clinical Research Center of Respiratory Diseases, No 2, East Yinghua Road, Chaoyang District, Beijing, 100029 China

**Keywords:** *Streptococcus gallolyticus subsp. pasteurianus*, Antibiotic resistance, Intrauterine infection

## Abstract

**Background:**

Infections by *Streptococcus gallolyticus subsp. pasteurianus* (SGSP) is often underestimated. Herein, the epidemiological features and resistant characteristics of SGSP in mainland China are characterized to enable a better understanding of its role in clinical infections.

**Methods:**

In the present work, 45 SGSP isolates were collected from the samples of bloodstream, urine, aseptic body fluid, and fetal membrane/placenta from patients in 8 tertiary general hospitals of 6 cities/provinces in China from 2011 to 2017. The identification of all isolates was performed using traditional biochemical methods, 16S rRNA and *gyrB* sequencing, followed by the characterization of their antibiotic resistance profiling and involved genes.

**Results:**

Among 34 non-pregnancy-related patients, 4 (4/34,11.8%) patients had gastrointestinal cancer, 10 (10/34, 29.4%) patients had diabetes, and one patient had infective endocarditis. Moreover, 11 cases of pregnant women were associated with intrauterine infection (9/11, 81.2%) and urinary tract infection (1/11, 9.1%), respectively. Except one, all other SGSP isolates were correctly identified by the BD Phoenix automated system. We found that all SGSP isolates were phenotypically susceptible to penicillin, ampicillin, cefotaxime, meropenem, and vancomycin. Forty strains (40/45, 88.9%) were both erythromycin and clindamycin-resistant, belonging to the cMLS_B_ phenotype, and the majority of them carried *erm*(B) gene (39/40, 97.5%). Although the cMLS_B_/*erm*(B) constituted the most frequently identified phenotype/genotype combination (25/40, 62.5%) among all erythromycin-resistant cMLS_B_ isolates, *erm*(B)/*erm*(A), *erm*(B)/*mef*(A/E), and *erm*(B)/*erm*(T) was detected in 7, 4, and 3 isolates, respectively. Furthermore, 43 strains (43/45, 95.6%) were tetracycline-resistant, and out of these, 39 strains (39/45, 86.7%) carried *tet*(L), 27(27/45, 60.0%) strains carried *tet*(O), and 7 (7/45, 15.6%) strains carried *tet*(M), alone or combined, respectively. All erythromycin-resistant isolates were also resistant to tetracycline.

**Conclusions:**

It is important to study and draw attention on SGSP, an underreported opportunistic pathogen targeting immunodeficient populations, notably elderly subjects, pregnant women and neonates.

## Background

*Streptococcus gallolyticus subsp. pasteurianus* (SGSP), formerly known as *S. bovis* biotype II/2 [1] and is one member of Group D streptococci, is a cause and a potential pathogen of bacteremia and infective endocarditis (IE), as well as urinary tract infection (UTI), in elderly and immunodeficient people [2–4], septicemia and meningitis in newborns, and as well as intrauterine infection in pregnant woman [5–8]. This species is also associated with gastrointestinal malignancy [3, 9]. It colonizes the digestive and female genital tract and therefore can lead to UTI and neonatal invasive infection, resembling what happens with group B *Streptococcus* (*S. agalactiae*, GBS). However, frequently occurring erroneous identification of SGSP might lead to an underestimation of the real incidence of infections caused by the species [6, 10]. Additionally, the susceptibility of SGSP strains to β-lactam and vancomycin has remained relatively stable over the past years, while variable resistance rates were observed against clindamycin, erythromycin, tetracycline and levofloxacin [4, 11, 12].

Considering gradually increased clinical infections caused by SGSP [10, 11], the clarification of its clinical features and antibiotic resistance is highly desired and should be valuable for its prevention and treatment. Unfortunately, epidemiological studies on SGSP isolates circulating in mainland China have not been conducted yet. To this end, we retrospectively analyzed SGSP isolates collected from 8 tertiary teaching hospitals in 6 cities/provinces in China from 2011 to 2017, and wanted to properly group these strains into species/subspecies level using traditional biochemical methods and 16S rRNA as well as *gyrB* sequencing to obtain their phenotypic and genotypic antibiotic resistance traits. The clinical and antibiotic resistance features of these SGSP isolates would help to understand the infections caused by the species circulating in China and for decision making in the context of empiric therapy.

## Methods

### Sample sources

Forty-seven non-duplicate isolates that were originally identified as SGSP in line with the new taxonomy criteria [13, 14] were recovered from 8 tertiary hospitals in China from 2011 to 2017, namely, Civil Aviation General Hospital (CAGH, Beijing) during 2011–2017, Affiliated Hospital of Inner Mongolia Medical University (Huhehot, Inner Mongolia (Neimenggu) Autonomous region) during 2016–2017, Henan Provincial People’s Hospital (Zhengzhou, Henan Province) during 2013–2017, Wuhan PuAi Hospital of Huazhong University of Science and Technology (Wuhan, Hubei Province) during 2014–2016, People’s Hospital of Guangxi Zhuang Autonomous Region (Nanning, Guangxi Zhuang Autonomous Region) during 2013–2017, Beijing Tsinghua Changgung Hospital, Medical Center of Tsinghua University (Beijing) during 2014–2016, Tai’an City Central Hospital (Tai’an, Shandong Province) in 2016, and Zhengzhou Children’s Hospital (Zhengzhou, Henan Province) in 2017.

### Phylogenetic analysis of 16S rRNA gene

DNAs were extracted from SGSP stains and subjected to PCR amplification and sequencing using a commercial DNA purification kit (Promega) according to the manufacturer’s instructions. The 16S rRNA genes from all SGSP strains were amplified with two universal primers (27F and 1492R), and the amplification of the DNA gyrase subunit B (*gyrB*) gene was performed using primers *gyrB* F 5′-GAAGTDGTIAARATYACBAAYCG-3′ and *gyrB* R5′-ACATCDGCATCRGTCAT-3′ as described elsewhere [15]. The sequencing of the 16S rRNA and *gyrB* was conducted by Ruibiotech (Beijing, China). The consequent comparison of the respective 16S rRNA and *gyrB* sequences against those in GenBank was performed using online BLASTn (www.ncbi.nlm.nih.gov/blast). A sequence similarity of 99 and 96% was used as a “cut-off” value for SGSP species identification [16, 17]. In addition, the phylogenetic tree was generated based on the 16S rRNA gene using the neighbour-joining algorithms using MEGA version 10.0.5 and iTOL v4 (https://itol.embl.de). To this end, the sequences were aligned with reference sequences of SGSP type strain AJ297216.1 that is available in the GenBank database.

### Bacterial identification using BD Phoenix automated microbiology system

**Fig. 1:**
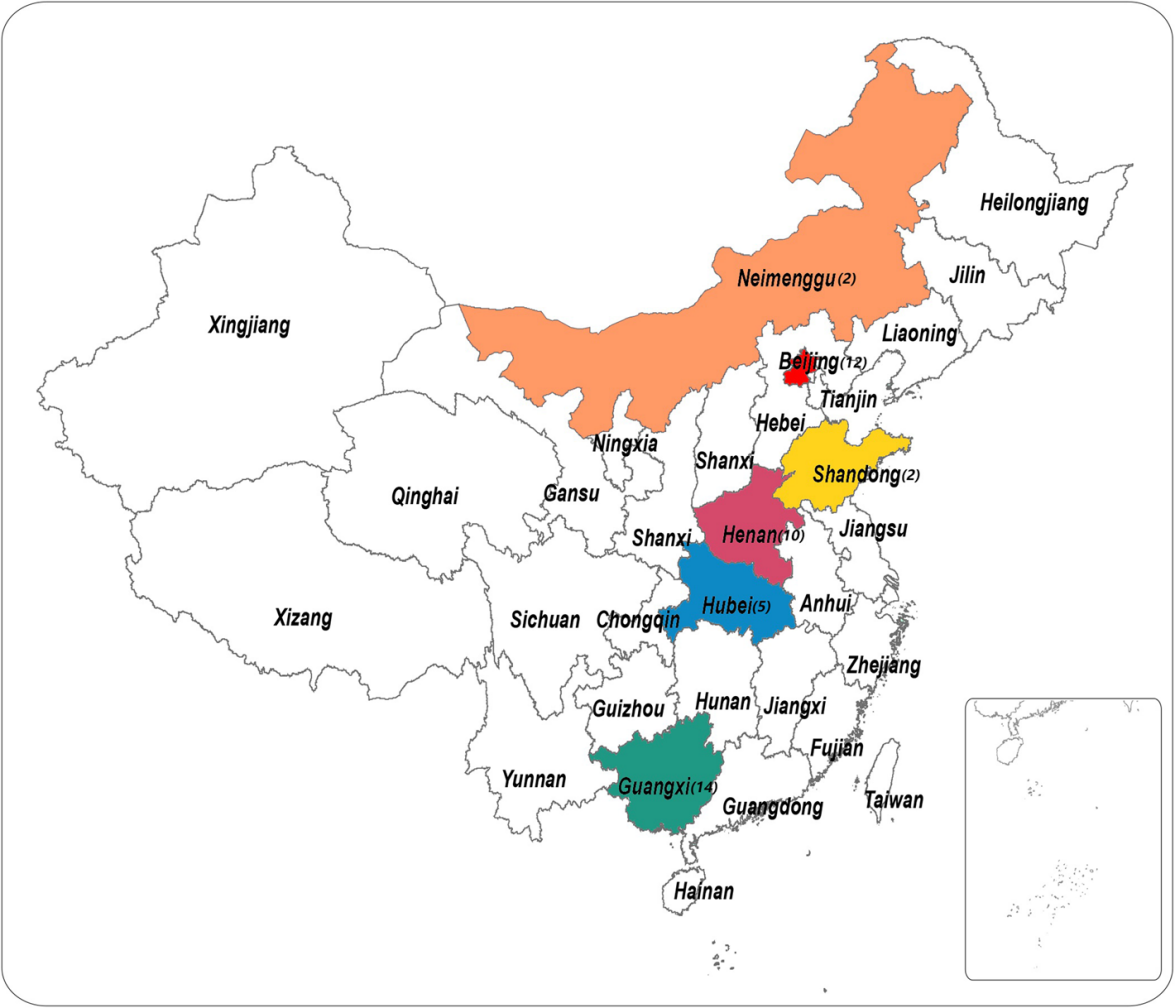
Geographical locations and numbers of *Streptococcus gallolyticus subsp. pasteurianus* (SGSP) isolates. The colored provinces represent where SGSP strains were isolated, with the number of isolates shown in brackets

All 47 isolates were examined by the department of clinical microbiology of CAGH for further confirmation based on the new taxonomy. BD Phoenix 100 Automated Microbiology System STREP (SMIC/ID) panel (Becton Dickinson, Sparks, MD, USA) was used as the identification method. The misidentified isolates by BD Phoenix 100 system, including *S. infantarius* (now designated as *S. infantarius* subspecies *infantarius*) (1 isolate) and *Enterococcus faecalis* (1 isolate), were excluded for further analysis. Finally, 45 SGSP isolates were included in the subsequent study and the clinical data of the patients are shown in Table 1. Their detailed geographic distribution was shown in Fig. 1.

**Table 1:** Demographic and clinical features of 45 isolates of *Streptococcus gallolyticus subsp. pasteurianus* circulating in mainland China

Infection types	Gender	Age	Sources	Underlying diseases	Polymicrobial	City/Province	Year of isolation
Non-pregnancy-related infections							
Abdominal infection	Female	41–60	Ascitic fluid	Choledochal cyst	No	Beijing	2016
Bacteremia(13)	Female(4), Male(9)	≤20, 21–40(2), 41–60(2), > 60(8)	Blood (13)	Hematuria, aplastic anemia, hematopoietic stem cell transplantation, endometrial carcinoma/colon cancer, fatty liver, cholecystitis, liver cancer, bone pain, abdominal pain, diabetes(3), ALL(2).	*Enterococcus faecalis*, MRSA.	Henan(2), Guangxi(5), Hubei(3), Shangdong, Neimenggu(2).	2014(3), 2015(2),2016(5),2017(3)
Bacteremia/infective endocarditis	Male	41–60	Blood		No	Guangxi	2015
Biliary infection	Female	> 60	Bile	Malignant bile duct tumor	No	Beijing	2013
Intra-abdominal infection	Male	> 60	Abdominal puncture fluid		No	Hubei	2014
Meningitis	Male	≤20	CSF	Anemia, pneumonia, congenital heart disease	No	Henan	2017
Peripancreatic abscess	Female	41–60	Peripancreatic drainage	Pancreatic tumor	No	Beijing	2015
UTI(15)	Female (10), Male (5)	21–40(2), > 60(13)	Urine (15)	Diabetes (7), hematuria, left renal calculus, hydronephrosis.		Beijing(2), Guangxi(5), Henan(5), Shangdong, Henan (2).	2011,2012(2), 2013(2), 2014, 2016(3),2017(6)
Pregnancy-related infections							
Bacteremia	Female	21–40	Blood	Delivery	No	Hubei	2014
Intrauterine infection(7)	Female (7)	21–40(7)	Fetal membrane (3), Fetal membrane/placenta (4).	Premature delivery, post-cesarean delivery, delivery (5)	No	Beijing(6), Guangxi	2011, 2012, 2013, 2014(2), 2015(2)
Intrauterine infection/bacteremia(2)	Female (2)	21–40(2)	Fetal membrane/blood (2)	Post-cesarean delivery	MRSA	Beijing, Guangxi	2012, 2017
UTI	Female	21–40	Urine		No	Guangxi	2017

*F* female, *M* male, *CSF* cerebrospinal fluid, *UTI* urinary tract infection, indicating symptomatic patients with bacteriuria, *ALL* Acute Lymphocytic Leukemia, *MRSA* methicillin-resistant *Staphylococcus aureus*; Number in parentheses represents strains, and no number signified only one strain was detected

### Phenotypical and genotypical features of antibiotic resistance

Susceptibility tests against penicillin, cefotaxime, vancomycin, meropenem, erythromycin, clindamycin, and tetracycline were performed using STREP (SMIC/ID) panel. The interpretive criteria for antibiotic susceptibility test (AST) were according to the Clinical and Laboratory Standards Institute (CLSI, 2017) for *Streptococcus spp. viridans* group.

Strains that showed resistant to erythromycin and tetracycline were also analyzed by PCR using conditions as described previously [1, 18], to examine the presence of antibiotic resistance genes, which are commonly found among the isolates of *S. bovis* group, including *erm*(A), *erm*(B), *erm*(T), *mef*(A/E), *tet*(K), *tet*(L), *tet*(M) and *tet*(O).

### Statistical analysis

In the study, MIC50 and MIC90 are defined as the MICs of a given agent that inhibits the growth of 50 and 90% of the isolates, respectively. MIC data of each antibiotic were recorded and analyzed by WHONET 5.6 software, and MIC50 and MIC90 were also calculated. Furthermore, the distribution of SGSP, as well as ages and infection types, was determined by using GraphPad Prism version 8.0.1.

## Results

### Clinical data

**Fig. 2:**
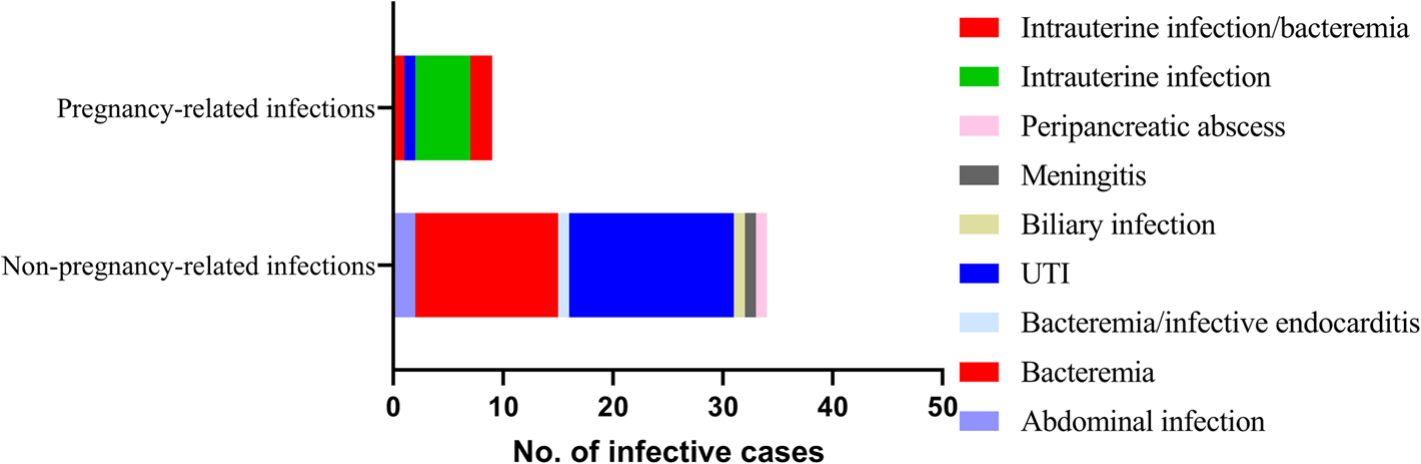
Distribution of the 45 strains of *Streptococcus gallolyticus subsp. pasteurianus* (SGSP) in different infections

**Fig. 3:**
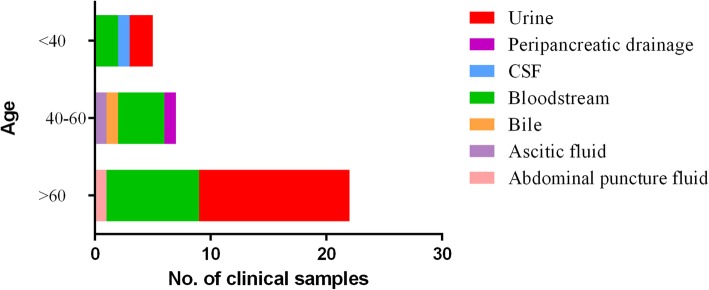
Relationship between infection sources and ages in 34 cases of non-pregnancy-related infections caused by *Streptococcus gallolyticus subsp. pasteurianus* (SGSP)

In the present study, the clinical data of these 45 patients with SGSP infections were reviewed and shown in Table 1 and Figs. 2 and 3. The majority of these patients were women (28/45, 62.2%). They were aged from 83 days to 87 years. There are 34 (75.6%) patients were non-pregnant, with an average age of 67 years old. Furthermore, the 45 SGSP isolates were obtained from bloodstream (17 cases, 37.8%, 2 cases were concurrently isolated from fetal membrane), urine (16 cases, 35.6%), bile (1 case, 2.2%), ascitic fluid (1 case, 2.2%), abdominal puncture fluid (1 case, 2.2%), peripancreatic drainage (1 case, 2.2%), peritoneal fluid (1 case, 2.2%), cerebrospinal fluid (CSF, 1 case, 2.2%) and fetal membrane/placenta (9 cases, 20.0%).

Among 34 non-pregnancy-related subjects, UTI and bacteremia accounted for 44.1% (15 cases) and 41.1% (14 cases), respectively (Fig. 3). The gender distribution was evenly distributed at a ratio of 1:1 (50%:50%). The majority of UTI cases occurred in patients over 60 years (13/16, 81.3%). Overall, 3 episodes out of 17 bacteremias were polymicrobial, where SGSP was simultaneously detected with Methicillin-resistant *Staphylococcus aureus* (MRSA) and *Enterococcus faecalis*.

Furthermore, in 34 non-pregnant patients, some underlying conditions had a higher prevalence: 10 patients (29.4%) had diabetes, 4 patients (11.8%) had presented with gastrointestinal cancers, and 1 patient (2.2%) had IE. One case was meningitis in a preterm male infant with late-onset infection (in his 83^rd^ day after born). This patient was born at 29^+ 2^ weeks with a birth weight of 1.45 kg. Moreover, 11 cases were associated with intrauterine infection (7 cases), bacteremia (1 case), or both (2 cases) in pregnancy-related infections.

### Strain identification and phylogenetic analysis of the 16S rRNA gene

**Fig. 4:**
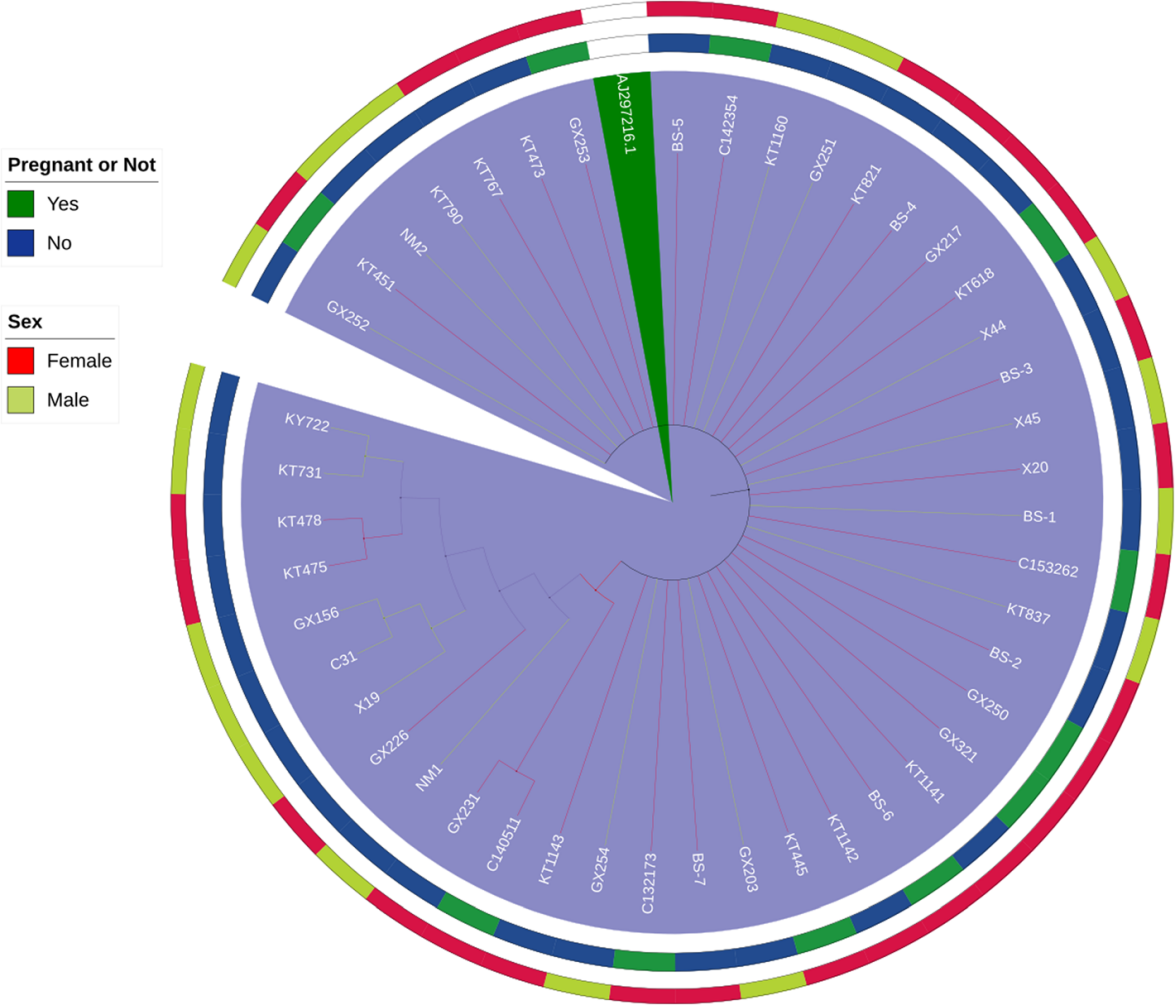
Phylogenetic tree constructed by the neighbour-joining method based on the nucleotide sequences of the 16S rRNA genes of 45 clinical *Streptococcus gallolyticus subsp. pasteurianus* (SGSP) strains and one reference strain AJ297216.1

All 45 isolates were positive for Streptococcus Lancefield antigen D grouping sera as examined by latex agglutination test. Initial identification by the automated Phoenix system revealed that all isolates belonged to *S. bovis* biotype II. Nucleotide sequencing of 16S rRNA amplicons classified all *S. bovis* biotype II isolates as SGSP. Furthermore, *gyrB* sequencing also identified the isolates as *S. pasteurianus*. The phylogenetic analysis of the 16S rRNA gene (1422 bp) was performed by the neighbour-joining method between the 45 SGSP strains and the reference strain of SGSP species (Fig. 4) [19].

### Antibiotic-resistant phenotypes and genotypes

Antimicrobial susceptibility results are shown in Table 2. All the isolates were phenotypically susceptible to penicillin, ampicillin, cefotaxime, meropenem, and vancomycin. Moreover, 41 strains (91.1%) showed a simultaneous resistance to erythromycin and clindamycin, and thus classified as cMLS_B_ phenotype. All erythromycin-resistant isolates carried at least an *erm*(B) gene, except KT478 strain, which was collected from peripancreatic abscess in a 57-year female, and harbored *mef*(A/E). No M phenotype or inducible MLS_B_ was detected. Among all erythromycin-resistant cMLS_B_ isolates, cMLS_B_/*erm*(B) phenotype/genotype was the most frequently identified combination (25 out of 40 strains, 62.5%), while *erm*(B)/*erm*(A), *erm*(B)/*mef*(A/E), and *erm*(B)/*erm*(T) were detected in 7, 4, and 3 isolates, respectively, as shown in Table 3.

**Table 2:** Antimicrobial susceptibilities and minimum inhibitory concentrations of 45 isolates of *Streptococcus gallolyticus subsp. pasteurianus*

Antimicrobials	Breakpoint by CLSI	Susceptibility			MIC		
		S(%)	I(%)	R(%)	MIC50 (mg/l)	MIC90 (mg/l)	Range (mg/l)
Penicillin	0.12/0.25–2/4	100	0	0	0.125	0.125	< 0.03–0.12
Amoxicillin	0.25/0.5–4/8	100	0	0	< 0.25	< 0.25	all < 0.25
Cefotaxime	1/2/4	100	0	0	< 0.5	< 0.5	all < 0.5
Erythromycin	0.25/0.5/1	11.1(5/45)	0	88.9(40/45)	> 4	> 4	0.0625- > 4
Clindamycin	0.25/0.5/1	11.1(5/45)	0	88.9(40/45)	> 4	> 4	0.0625~ > 4
Levofloxacin	2/4/8	73.3(33/45)	15.6(7/45)	11.1(5/45)	2	4	1~ > 4
Moxifloxacin	1/2/4	88.9(40/45)	2.2(1/45)	8.9(4/45)	0.5	1	≤0.25~ > 2
Tetracycline	2/4/8	4.4(2/45)	2.2(1/45)	93.3(42/45)	> 8	> 8	0.0625~ > 4
Linezolid	2	100	0	0	≤1	2	≤1~2
Meropenem	0.5	100	0	0	≤0.0625	≤0.0625	≤0.0625
Vancomycin	1	100	0	0	≤0.25	≤0.25	≤0.25–1

*MIC* minimum inhibitory concentration, *S* susceptible, *I* intermediate, *R* resistant, *MIC50* minimum inhibitory concentration at which 50% of isolates were inhibited, *MIC90* minimum inhibitory concentration at which 90% of isolates were inhibited, *MIC range* range of minimum inhibitory concentration

**Table 3:** Erythromycin and tetracycline resistance phenotype and genotype in 45 *Streptococcus gallolyticus subsp. pasteurianus* isolates

Erythromycin phenotype	Clindamycin MIC (μg/mL)	Erythromycin MIC (μg/mL)	Erythromycin resistance genotype	Tetracycline MIC (μg/mL)	Tetracycline phenotype	Tetracycline resistance genotype	No. of isolates
cMLS_B_	> 1	> 4	*erm*(A)/*erm*(B)	> 8	R	*tet*(M)/*tet*(L*)*	2
cMLS_B_	> 1	> 4	*erm*(A)/*erm*(B)	> 8	R	*tet*(M)/*tet*(O*)*	1
cMLS_B_	> 1	> 4	*erm*(A)/*erm*(B)	> 8	R	*tet*(L)/*tet*(O*)*	1
cMLS_B_	> 1	> 4	*erm*(A)/*erm*(B)	> 8	R	*tet*(O*)*	3
cMLS_B_	> 1	> 4	*erm*(B)	4	I	Negative	1
cMLS_B_	> 1	> 4	*erm*(B)	> 8	R	*tet*(L)	8
cMLS_B_	> 1	> 4	*erm*(B)	> 8	R	*tet*(L)/*tet*(O*)*	1
cMLS_B_	> 1	> 4	*erm*(B)	> 8	R	*tet*(M)/*tet*(L*)*	1
cMLS_B_	> 1	> 4	*erm*(B)	> 8	R	*tet*(M)/*tet*(O*)*	1
cMLS_B_	> 1	> 4	*erm*(B)	> 8	R	*tet*(O)	7
cMLS_B_	> 1	> 4	*erm*(B)	> 8	R	*tet*(L)/*tet*(O*)*	5
cMLS_B_	> 1	> 4	*erm*(B)	> 8	R	*tet*(O*)*/*tet*(L*)/tet*(M*)*	1
cMLS_B_	> 1	> 4	*erm*(B)/*mef*(A/E)	> 8	R	*tet*(L*)*	1
cMLS_B_	> 1	> 4	*erm*(B)/*mef*(A/E)	> 8	R	*tet*(O*)*/*tet*(L*)*	3
cMLS_B_	> 1	> 4	*erm*(B)/*erm*(T)	> 8	R	*tet*(M)/*tet*(O*)*	1
cMLS_B_	> 1	> 4	*erm*(B)/*erm*(T)	> 8	R	*tet*(O*)*/*tet*(L*)*	1
cMLS_B_	> 1	> 4	*erm*(B)/*erm*(T)	> 8	R	*tet*(L)	1
cMLS_B_	> 1	> 4	*mef*(A/E)	> 8	R	*tet*(O*)*/*tet*(L*)*	1
S	0.125	≤0.0625	Negative	> 8	R	*tet*(L)	1
S	0.0625	≤0.0625	Negative	> 8	R	*tet*(L)	1
S	0.0625	≤0.0625	Negative	> 8	R	*tet*(O*)*/tet(L*)*	1
S	0.0625	≤0.0625	Negative	≤0.5	S	Negative	2

cMLS_B_, constitutive macrolide-lincosamide-streptogramin B resistance; S/I/R: the isolates susceptible/intermediate/resistant to macrolide or tetracycline

Moreover, in 43 (95.6%) out of 45 were tetracycline-resistant strains, 29 strains harbored *tet*(L), 27 strains *tet*(O) and 7 strains *tet*(M), singly or combined. One isolate harbored triple resistance genes of *tet*(O)/*tet*(L)/*tet*(M) at the same time. No *tet*(K) gene was identified. Interestingly, all erythromycin-resistant isolates were also resistant to tetracycline, and both tetracycline-sensitive isolates were also sensitive to erythromycin.

### Literature review

To better understand the features of SGSP infections worldwide, we searched MEDLINE database (https://www.ncbi.nlm.nih.gov/pubmed) for the studies reporting the clinical infections caused by SGSP. Five reports including185 clinical SGSP isolates were included for comparison, and the details were summarized in Table 4 [4, 11, 14, 20, 21].

**Table 4:** Summary of the reported cases of clinical infections by *Streptococcus gallolyticus subsp. pasteurianus*

	present study	1 [4]	2 [11]	3[14]	4 [20]	5[21]
Number	45	20 patients (22 isolates were recovered)	126	24	13	2
Study period	2011–2017	May 2010-Jan 2012	2000–2012	January 2003 and January 2010	2004–2010	1988–2014
Country/area	China	Italy	Taiwan	Spain	Israel	Spain
Demographic characteristics						
Gender (Male/female)	17/28	7/13	79/47	NA	7/6	1/1
Age (mean ± SD or median and IQD, years)	67 ± 22	72 ± 13	70(55,78)	NA	60 ± 33	89 and 62
Age > 65 years	19(42.2%)	15	76(60%)	NA	NA	1(50%)
Pregnant women	11(24.4%)	0	NA	NA	2(15%)	0
Neonates(< 3 months of age)	1(2.2%)	0	NA	NA	2(15%)	0
Paediatric patients (< 18 years)	1(2.2%)	0	5(4%)	NA	NA	NA
Infection types/source	Bacteremia (17), urine (16), bile (1), ascitic fluid (1), abdominal puncture fluid (1), peripancreatic drainage (1), peritoneal fluid (1), CSF(1) and fetal membrane/placenta (9)	UTI(10), bacteremia(2), limb ulcer (1), bile(3)	Bacteremia(126)	Bacteremia(24)	Bacteremia(13)	Spondylodiscitis/ paravertebral abscess (1), pubicsymphysitis and UTI(1)
Underlying diseases						
Diabetes	10(22.2%)	8(40%)	43(34%)	2(8.3%)	2(15%)	1(50%)
Chronic renal failure	NA	1(5%)	22(17%)	1(4.2%)	3(23%)	NA
Liver disease	NA	4(20%)	53(42%)	NA	4(31%)	NA
Malignancy (past or active)	4(8.9%)	6(30%)	68(54%)	9(37.5%, colonic adenoma (4); bladder cancer(1); prostate cancer(1); pulmonary cancer(1); mucosa-associated lymphoid tissue lymphoma (1); leukemia(1))	3(23%)	Adenoma(1); prostate cancer(1)
Gastrointestinal tract tumors	4(8.9%)	4(20%)	51(40%, including colon/rectum, stomach, pancreas, liver, bile duct).	4(16.7%)	4(31%)	1(50%)
Biliary pathology	1(2.2%)	4(20%)	9(7%, biliary tract stone)	5(20.8%)	2(15%)	NA
Bacteriuria	16(35.6%)	14(70%)(50% UTI);	2(2%)	0	NA	1(50%)
Endocarditis	1(2.2%)	2(10%)	17(13%)	6(25%)	3(23%)	1(50%)
Identification methods	BD Phoenix 100, 16S rRNA/*gyrB* sequencing	Phoenix100, 16S rDNA sequencing, MALDI Biotyper Bruker and Vitek MS	Vitek automated system; 16S rRNA and sodA genes and PCR-RFLP assays of groESL gene	API 20 Strep system, semiautomated Wider system, 16S rRNA and *sodA* PCR, Bruker Biotyper MALDI-TOF MS	PCR-RFLP/Vitek 2	API 20 Strep system/ Vitek 2, 16S rRNA/*sodA* sequencing
Antibiotic susceptibility rate (median, μg/mL)(susceptibility rate(%))						
penicillin	0.125(100)	NA(100)	0.06(100)	NA (NA)	0.06(100)	NA (NA)
cefatriaxone	< 0.5(100)	NA(100% to cefotaxime)	0.12(100)	NA (NA)	0.09(100)	NA (NA)
erythromycin	> 4(11.1%, *erm*(B)*/erm*(A), *erm*(B)/*mef*(A/E), and *erm*(B)/*erm*(T) were detected in 7, 4, and 3 isolates, respectively).	NA(68.2%, all resistant isolates belonged to the cMLS_B_ phenotype, carried *erm*(B)).	32(37%)	0.5(62.5%)	NA (NA)	NA (NA)
clindamycin	> 4(11.1%)	NA(68.2%)	0.06(39%)	0.5(75%)	NA (NA)	NA (NA)
levofloxacin	2(73.3%)	NA (NA)	2(82%)	0.5(91.7%)	NA (NA)	NA (NA)
vancomycin	≤0.25	NA (NA)	0.5(100%)	NA (NA)	NA (NA)	NA (NA)
tetracycline (μg/mL)	> 8((4.4%), 39 strains carried *tet*(L), 27 *tet*(O), and 7 *tet*(M), alone or combined, respectively.)	NA(31.8%, the resistant isolates carried *tet*(O)(11) and *tet*(M)(4).	NA (NA)	NA (NA)	NA (NA)	NA (NA)

*NA* Not available, *CSF* Cerebrospinal fluid, *UTI* urinary tract infection; Number in parentheses represents strains, and no number signified only one strain was detected

## Discussion

This work is, to the best of our knowledge, the first comprehensive study on infective SGSP isolates in mainland China. The complexity of *S. bovis* taxonomy and relatively limited infection reports constrain clinical studies of SGSP, which is thus considered as an underreported opportunistic pathogen [10, 14]. In the present study, we found that 22 out of 34 non-pregnancy-related subjects (64.7%) were elderly subjects with ages over 65 years. Interestingly, a study conducted in southern-central Israel reported that 75% bacteremia by *S. bovis* was over 65 years [20]. This, together with our data, suggests that elderly people are prone to SGSP infection. Moreover, we also observed that among 15 SGSP isolates (15/34, 44.1% non-pregnant subjects) recovered from UTI patients, 10 had diabetes. This observation was in line with a retrospective study in Italy, which reported that among 63.6% of patients (14/22) with UTI caused by SGSP, diabetes was the most common underlying disease (7/22, 31.8%) [4]. Another study in Spain also found that most *S. bovis* group isolates (72%) causing UTI were SGSP [2]. These observations thus collectively hint that SGSP can be taken as a potential pathogen in UTI, especially in those with diabetes [2]. Additionally, it should be noted that 62.5% patients (10/16) with SGSP bacteremia were male, demonstrating a correlation between gender and SGSP isolation from the urinary tract, as suggested in another two previous studies [4]. Together, the elderly, pregnant women and the immunodeficient population are the main people who are under the risk of SGSP infection.

Bacteremia caused by SGSP was shown to be associated with malignancy of various parts of the digestive tract, including gastric, pancreatic, hepatobiliary and colorectal cancers [11, 22–25]. In our study, 1 case of malignant bile duct tumor, 1 case of endometrial carcinoma/colon cancer, 1 case of pancreatic tumor and 1 case of liver cancer were identified in non-pregnant-related infections, respectively. It was reported that all *S. bovis* strains from bile were likely associated with biliary tract malignancy [20], and SGSP was more frequently identified in the bacteremia with a biliary source (15/27 cases, 55.6%,) than *S. infantarius* (20/46, 43.5%) and *S. gallolyticus* subsp. *gallolyticus* (SGSG, 2/112, 1.8%) [23]. It should be noted, in colorectal carcinoma, a lower risk was noticed for SGSP, compared with SGSG at an odds ratio of 7.26 [26]. As a consequence, considering the association between *S. bovis* subspecies and specific pathogenesis, it is thus mandatory for every *S. viridans* organism isolated from the bloodstream to be identified into a species/subspecies level in order to distinguish SGSP from other *S. bovis* group members [4, 10, 22]. Additionally, SGSP bacteremia was observed to be less associated with IE than SGSG (8~29% in SGSP vs 43~100% in SGSG), too [22, 26]. Only one case (1/16, 6.25%) of SGSP bacteremia was diagnosed with IE in the present study. Another observation was that the hematological diseases, including aplastic anemia (with hematopoietic stem cell transplantation) and acute lymphoblastic leukemia, were detected in two patients in our study, and this has been rarely documented previously [22]. The underlying mechanism remains elusive.

SGSP colonizes asymptomatically in the gastrointestinal and genitourinary tracts in pregnant women, and thus might potentially cause neonatal meningitis and bacteremia [7, 8, 27, 28]. There is a very high one-year mortality rate of 58.7% in SGSP bacteremia [22]. The current study involves 9 cases (9/45, 20%) of intrauterine infections, and 2 cases (2/45, 4.4%) of bacteremia in pregnant women and neonates, hinting that SGSP is an important pathogen of pregnancy-related infection [8, 28–30]. One case of late-onset meningitis in a preterm male infant is detected in this study. Furthermore, our previous report found one case of intrauterine infection and post-partum bacteremia that was attributed to SGSP providing evidence of a possible portal of entry in cases of maternal or neonatal infection [8]. This potential infective pathway might be confirmed because more similar cases exist in this work. Altogether, we support the hypothesis that SGSP, which is different from other subspecies of *S.bovis* group, is a potential pathogen of maternal-fetal infection similarly to GBS [8].

Phenotypic variations always limit a correct identification of *S. bovis* species by the use of conventional microbiology and biochemical methods. However, in this study, Phoenix100 system identified most SGSP strains into subspecies level correctly, except the KT445 strain collected from fetal membrane in a 32-year female, and that was misidentified as *S. bovis* I (Strep. group D) by Phoenix100, but confirmed as SGSP (*S. pasteurianus* strain CIP 107122) using 16S rRNA gene. Therefore, it is tempting to conclude that the classical biochemical methods are suitable and sufficient to fulfill clinical purposes.

Treatment of SGSP infection, especially in meningitis, often includes intravenous penicillin, ampicillin and cefotaxime administration [7]. Considering all SGSP isolates were susceptible to penicillin [2], cefotaxime, vancomycin, meropenem, and chloramphenicol, the narrowest spectrum antibiotic penicillin should be considered as the drug of choice. In line with our results, this antibiotic choice should be recommended in mainland China. Furthermore, SGSP resistance rates varied for clindamycin, erythromycin, tetracycline and levofloxacin [11]. In our study, most SGSP isolates (40/45, 88.9%) simultaneously exhibited resistance to macrolides and clindamycin, dramatically higher than that of 31.8% in Italy [4] and 37.5% in Spain [14]. The resistance of SGSP isolates was due to the presence of either *erm*(B) and *erm*(T) genes or to a lesser extent *mef*(A/E) gene [4, 31, 32]. All erythromycin-resistant isolates in this study also displayed resistance to clindamycin, with the cMLS_B_ resistance phenotype caused mainly by the *erm*(B) gene. While efflux-encoding *mef*(A/E) genes were only detectable in 5 isolates, singly (1 case) or combined with *erm*(B) (4 cases), which is different from previous reports in which *erm*(T) was found to be responsible for most macrolide resistance [12, 31]. Among the reported SGSP strains in Italy, 68.2% (15/22) were tetracycline-resistant, and most of them harbored either *tet*(O) (10 cases) or *tet*(M) (4 cases) [4]. In the current study, however, 93.3% strains were tetracycline-resistant, most carried *tet*(L) gene, and less carried *tet*(O) and *tet*(M) genes, singly or combined, while no isolate carried *tet*(K) gene. This discrepancy might be explained by geographic and/or species differences. Furthermore, all erythromycin-resistant isolates were also resistant to tetracycline, similar to a previous study [4] . Taken together, these findings demonstrated that antibiotic resistance was widespread among SGSP clinical isolates, thus representing a serious problem particularly when the emerging infection rates are considered, especially in those allergic to β-lactam antibiotics.

## Conclusions

In summary, this study on infective SGSP isolates circulating in mainland China underscores the clinical importance of this microorganism and provides valuable information about clinical features and epidemiological characteristics of SGSP. It is important to draw attention to this underreported opportunistic pathogen targeting immunodeficient populations, notably elderly subjects, pregnant women and neonates.

## Data Availability

I can confirm I have included a statement regarding data and material availability in the declaration section of my manuscript. All the data and material involved in the current study are available from the corresponding author on reasonable request.

## Abbreviations

IE: infective endocarditis
MRSA: Methicillin-resistant *Staphylococcus aureus*
SGSG: *Streptococcus gallolyticus* *subsp.* *gallolyticus*
SGSP: *Streptococcus gallolyticus subsp. pasteurianus*
UTI: urinary tract infection
